# Optimization of physical schemes in WRF model on cyclone simulations over Bay of Bengal using one-way ANOVA and Tukey’s test

**DOI:** 10.1038/s41598-021-02723-z

**Published:** 2021-12-24

**Authors:** Meenakshi Shenoy, P. V. S. Raju, Jagdish Prasad

**Affiliations:** 1 Centre for Ocean Atmospheric Science and Technology, Amity University Rajasthan, Kant Kalwar, Jaipur, Rajasthan India; 2 Amity School of Applied Sciences, Amity University Rajasthan, Kant Kalwar, Jaipur, Rajasthan India

**Keywords:** Natural hazards, Statistics

## Abstract

Evaluation of appropriate physics parameterization schemes for the Weather Research and Forecasting (WRF) model is vital for accurately forecasting tropical cyclones. Three cyclones Nargis, Titli and Fani have been chosen to investigate the combination of five cloud microphysics (MP), three cumulus convection (CC), and two planetary boundary layer (PBL) schemes of the WRF model (ver. 4.0) with ARW core with respect to track and intensity to determine an optimal combination of these physical schemes. The initial and boundary conditions for sensitivity experiments are drawn from the National Centers for Environmental Prediction (NCEP) global forecasting system (GFS) data. Simulated track and intensity of three cyclonic cases are compared with the India Meteorological Department (IMD) observations. One-way analysis of variance (ANOVA) is applied to check the significance of the data obtained from the model. Further, Tukey’s test is applied for post-hoc analysis in order to identify the cluster of treatments close to IMD observations for all three cyclones. Results are obtained through the statistical analysis; average root means square error (RMSE) of intensity throughout the cyclone period and time error at landfall with the step-by-step elimination method. Through the elimination method, the optimal scheme combination is obtained. The YSU planetary boundary layer with Kain–Fritsch cumulus convection and Ferrier microphysics scheme combination is identified as an optimal combination in this study for the forecasting of tropical cyclones over the Bay of Bengal.

## Introduction

Tropical cyclones are mesoscale disturbances originating in the warm tropical Oceans. High sea surface temperatures, low atmospheric pressure, weak wind shear and high mid-tropospheric moisture favor the formation and intensification of tropical cyclones. The heavy precipitation, strong winds and associated storm surge are devastating over the coastal regions during and after landfall. Study of cyclones over Bay of Bengal from 1891 to 2010, depicted an increase in the number of severe cyclonic storms in the Bay of Bengal^1^. Balaji et al. in 2018^2^ points that the shallow bathymetry, low lying coastal terrain, funnel shape of coastline and coastal population density accentuates the devastation during landfall.

Advanced Weather Research and Forecast (WRF) model is one of the state-of-the art numerical weather prediction (NWP) model, which serves as a vital tool for forecasting of tropical cyclones. The WRF model with ARW core have been used by numerous researchers to study the impact of model physics on track and intensity of cyclones over the north Indian ocean^3–8^. Various physics schemes namely, the planetary boundary layer (PBL), cumulus convection (CC) and cloud microphysics (MP), longwave and shortwave radiation, and land-surface schemes are employed during forecasting. Sensitivity studies have been conducted to identify the combination of suitable physics schemes for prediction of track and intensity of tropical cyclones over Bay of Bengal^9–16^. Those studies identify the schemes based on the bias in the mean square error and average track error. However, in the present study a methodology is developed using one-way analysis of variance (ANOVA)^17^ to test the significant difference among the various combination of schemes for intensity and track prediction over three severe cyclones of the Bay of Bengal (BoB). Further, Tukey’s post-hoc test^18^ is applied to identify the pairs of combination of schemes which are significantly close to IMD observations. From this process, the treatment combinations which are significantly different from IMD observations are eliminated and thus four cluster of treatments with respect to four variables (central pressure, maximum sustained wind, longitude and latitude) are obtained for every cyclone. These clusters of scheme combinations are then tested for the error at landfall time and average Root Mean Square Error (RMSE) of central pressure (CP) and maximum sustained wind (MSW) with respect to three cyclones and through elimination process optimal scheme is obtained.

## Experimental design

In order to obtain reasonable optimal combination of schemes of WRF model, three severe cyclonic cases over Bay of Bengal (Nargis-2008; Titli-2018 and Fani-2019) are considered in this study. These cyclones are severe cyclones considered as a sample for the entire population of tropical cyclones in the Bay of Bengal. The optimization method tested for the sample can then be applied over the population. The sensitivity experiments are carried out with the combination of two PBL schemes (Yonsei University scheme and Mellor–Yamada–Janjic), three CC schemes (Kain–Fritsch, Betts–Miller–Janjic and Grell–Devenyi) and five MP schemes (Ferrier, WSM6, Thompson, Lin and Kessler) on three cyclonic cases. Thus, a total of 90 sensitivity experiments (2 PBL × 3 CC × 5 MP × 3 TCs) are performed using Weather Research and Forecasting model (WRF) with ARW core, version 4.0 (Table 1). The detailed description of WRF model physics and dynamics are given by Skamarock et al.^19^. The initial and boundary conditions are obtained from the National Centers for Environmental Prediction (NCEP) Global Analysis and Forecasting System (GFS) data available at 0.5° × 0.5° resolution, with time varying boundary conditions updated at every 6 h interval. The model is integrated over Bay of Bengal (4.37° S–27.44° N and 68.22°–107.78° E) having Mercator map projection with 9 km horizontal resolution and 35 vertical levels. The Arakawa C-grid grid staggering is used along with third-order time integration scheme and sixth-order advection scheme in both horizontal and vertical directions. Dudhia scheme^20^ and the Rapid Radiative Transfer Model (RRTM) scheme^21^ are used for short wave and long-wave parameterization respectively in all the sensitivity experiments. For the surface parameterization, the Noah land-surface scheme is considered. For all three cyclones, the model is initialized three days prior to the cyclone landfall and integrated upto 120 h. i.e. initial condition for cyclones Nargis, titli and Fani are considered respectively 28april2008-12UTC, 08oct2018-12UTC and 30april2019-12UTC The observed cyclone track and intensity from the India Meteorological Department (IMD) are used to estimate the track and intensity errors. In this study, central pressure (CP; hPa) and 10-m maximum sustained wind (MSW; ms^−1^) are used to estimate the intensity of tropical cyclone.

**Table 1:** Physical parameterization schemes selected for the combinatorial optimization.

Code number of PBL	Planetary boundary layer scheme (PBL)	Code number of CC	Cumulus convection scheme (CC)	Code number of MP	Cloud microphysics scheme (MP)
1	Yonsei University Scheme (YSU)	1	Kain-Fritsch (KF)	1	Ferrier
2	Mellor-Yamada-Janjic (MYJ)	2	Betts-Miller-Janjic (BMJ)	2	WSM6
		3	Grell-Devenyi (GD)	3	Thompson
				4	Lin
				5	Kessler

In this optimality study, the best performing combination of physics schemes have been investigated individually in relation to time at landfall, intensity (CP, MSW) and track (latitude, longitude) along the trajectory of cyclone. The physics schemes as well as their codes are presented in Table 1. The treatments are coded from 0 to 30, ‘0’ being the IMD observation. Treatment 1–30 are combinations ordered by considering the PBL scheme at first followed by CC scheme and MP scheme viz. treatment 1 will be a combination of first PBL-YSU with first CC-KF and MP Ferrier. The treatments 1 to 5 are formed by keeping the first PBL and first CC scheme constant and orderly varying the five MP schemes listed in Table 1. The treatments 6–10 are created with YSU PBL, second CC scheme viz. BMJ and varying the MP schemes. Similarly, all the other treatment combinations are created. The means of 31 treatments are compared in order to check the variability in the average effect of treatments using one-way ANOVA. Further, groups of treatments which provide values significantly close to treatment ‘0’ are determined using Tukey’s post-hoc test, while the other treatments are eliminated. The RMSE of CP and MSW are computed every 6 h and averaged over the entire duration of cyclone. The clusters of treatments obtained from the Tukey’s test are checked for RMSE and time error at landfall. The treatment which is consistently close to treatment 0 (IMD observations) for all the variables (CP, MSW, longitude and latitude) with respect to three cyclones with the elimination procedure, is termed as the optimal combination of scheme.

## Results

### Titli

**Figure 1:**
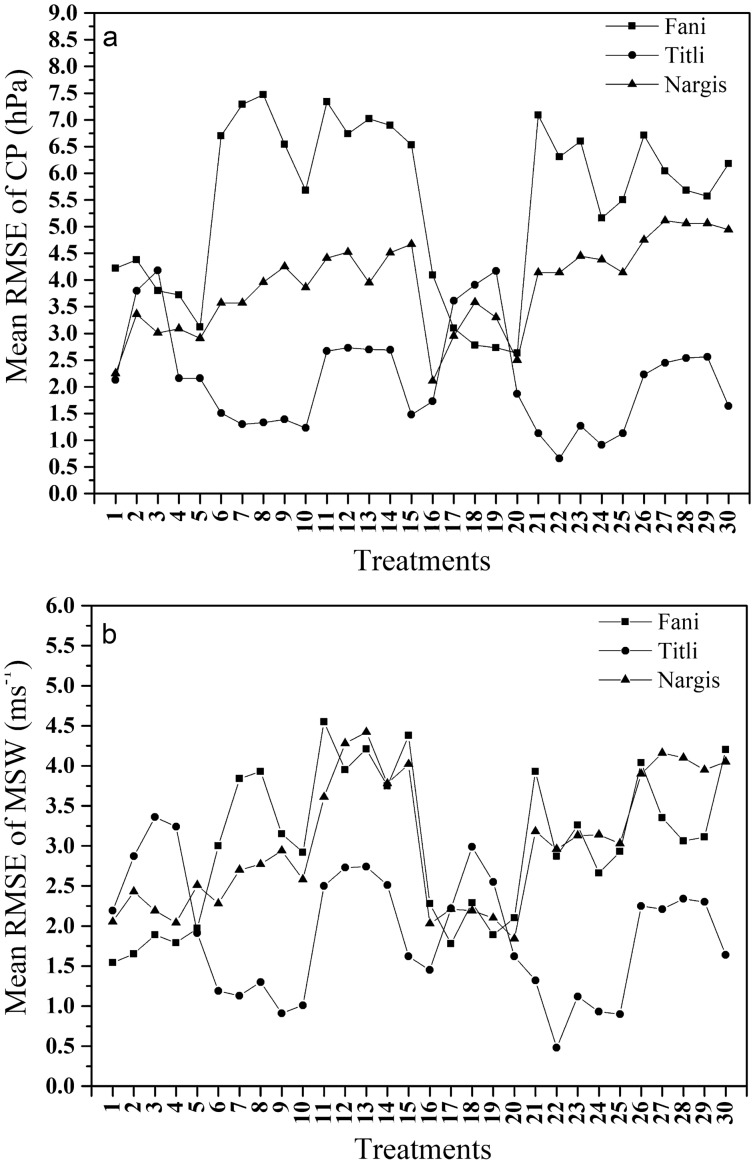
Root mean square (RMS) error averaged for the entire simulation period for (**a**) central pressure and (**b**) MSW for cyclone Fani, Titli and Nargis.

**Figure 2:**
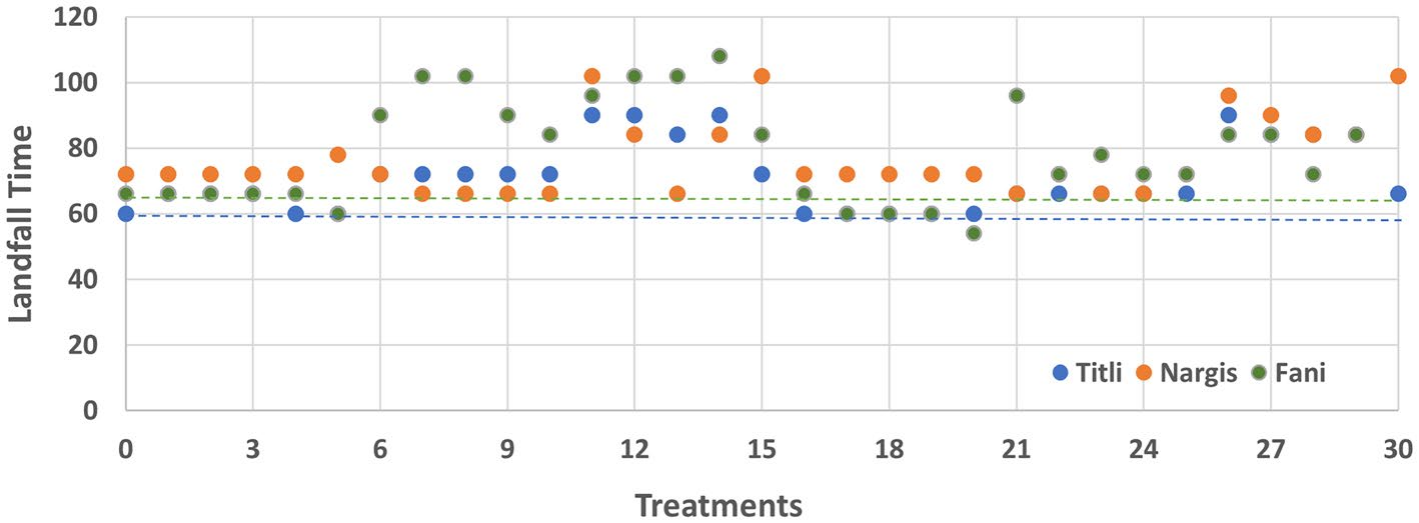
Landfall time for three cyclones Fani, Titli, and Nargis for different combination of schemes.

One-way ANOVA provides results from which we can identify whether the average effect of treatments is similar to one another. For cyclone Titli, this analysis (Tables 2 and 3) resulted in rejecting the null hypothesis for intensity variables CP (F (30, 527) = 1.434, p = 0.066) and MSW (F (30, 527) = 3.107, p = 0.00). Similarly, the average effect of treatments vary from each other for variables Longitude (F (30, 527) = 1.607, p = 0.023) and Latitude (F (30, 527) = 2.710, p = 0.00) (Tables 4 and 5). For Titli, the group of treatments significantly close to treatment 0 for CP are 16, 9, 6, 12, 14, 1, 4, 5, 20 and 13; and MSW are treatments 10, 29, 28, 24, 22, 27, 1, 19, 2, 18, 4 and 3 respectively (Tables 14b and 15b). The treatments that are much the same as IMD observations are treatments 1, 12, 28, 14 and 3 for longitude and treatments 1, 4, 2, 3, 16 and 23 for latitude variable respectively. The treatments 1, 3 and 4 are consistently similar to treatment 0 with respect to all the variables. The root means square error (RMSE) was computed every six hourly and averaged over the entire simulation period for intensity variables. The treatments which were inconsistent in the intensity, and track variables have been disregarded when considering the treatments considerably near to treatment 0 for RMSE (CP and MSW) and time error at landfall. It is observed that treatments 1 and 3 with respect to CP while, treatment 1 for MSW provides least RMSE (2.13–2.91 hPa; 4.18 ms^−1^) (Fig. 1). Similarly, treatments 1 and 4 have time of landfall substantially close to treatment 0 (Fig. 2). Thus, for cyclone Titli, treatment 1 i.e., YSU-KF-Ferrier is obtained as the optimal combination.

**Table 2:** One-way ANOVA for Titli longitude.

	Sum of squares	df	Mean square	F	Sig
Between groups	263.644	30	8.788	1.607	.023
Within groups	2882.577	527	5.470		
Total	3146.221	557

**Table 3:** One-way ANOVA for Titli latitude.

	Sum of squares	df	Mean square	F	Sig
Between groups	174.841	30	5.828	2.710	0.000
Within groups	1133.290	527	2.150		
Total	1308.130	557

**Table 4:** One-way ANOVA for Titli CP.

	Sum of squares	df	Mean square	F	Sig
Between groups	13,314.182	30	443.806	1.434	0.066
Within groups	163,084.982	527	309.459		
Total	176,399.164	557

**Table 5:** One-way ANOVA for Titli MSW.

	Sum of squares	df	Mean square	F	Sig
Between groups	9196.901	30	306.563	3.107	0.000
Within groups	52,005.124	527	98.681		
Total	61,202.026	557

### Fani

In case of cyclone Fani, the null hypothesis is rejected for the average effect of the treatments with respect to Longitude, latitude, CP and MSW (Tables 6, 7, 8, 9). The mean effect of treatments significantly vary for these variables at (F (30, 465) = 1.423, p = 0.071); (F (30, 527) = 1.401, p = 0.080) and (F (30, 527) = 1.875, p = 0.004). Whereas, Table 7 depicts that the average effect of treatments for latitude are similar to each other at (F (30, 527) = 0.804, p = 0.762). The treatments 20, 18, 19, 1, 5, 17 and 2 are close to IMD observations for CP whereas, for MSW the treatments 1, 2, 4, 17, 3, 19 and 10 are significantly close to treatment 0 (Table 14a). For latitude variable it is seen that treatments close to IMD observations are 13, 21, 1, 8, 2 and 7; and that for longitude are 5, 20, 17, 18, 2 and 1 respectively (Table 15a). The treatments that are not in compliance for all the variables (intensity and track) are being overlooked. From the group of consistent treatments, treatment 1 and 2 result in lower values of RMSE for both CP (4.22–4.38 hPa) and MSW (1.54–1.65 ms^−1^) respectively (Fig. 1). The same result follows for landfall time error for cyclone Fani from Fig. 2. In Tilti treatment 1 is optimal and the same result is consistent in Fani.

**Table 6:** One-way ANOVA for Fani longitude.

	Sum of squares	df	Mean square	F	Sig
Between groups	73.906	30	2.464	1.875	0.004
Within groups	651.645	496	1.314		
Total	725.551	526

**Table 7:** One-way ANOVA for Fani latitude.

	Sum of squares	df	Mean square	F	Sig
Between groups	284.629	30	9.488	0.804	0.762
Within groups	5850.948	496	11.796		
Total	6135.577	526

**Table 8:** One-way ANOVA for Fani CP.

	Sum of squares	df	Mean square	F	Sig
Between groups	12,713.008	30	423.767	1.423	0.071
Within groups	138,475.435	465	297.797		
Total	151,188.443	495

**Table 9:** One-way ANOVA for Fani MSW.

	Sum of squares	df	Mean square	F	Sig
Between groups	3473.020	30	115.767	1.401	0.080
Within Groups	38,421.998	465	82.628		
Total	41,895.018	495			

### Nargis

Tables 10, 11, 12 and 13 provides ANOVA results for cyclone Nargis. The null hypothesis is rejected for variables CP (F (30, 527) = 2.272 p = 0.00), MSW (F (30, 527) = 4.899, p = 0.00) and Latitude (F (30, 465) = 1.658, p = 0.017). Whereas, the null hypothesis is accepted for longitude at F (30, 465) = 1.658, p = 0.595. The Tukey’s test for CP variable suggests treatments 18, 2, 3, 19, 4, 5, 17 and 1; whereas, treatments 2, 18, 3, 5, 4, 17, 19 and 1 for MSW are significantly analogous to treatment 0. From Table 15c, the group of treatments 20, 5, 18, 2, 3, 19, 4, 16, 25, 6, 17, 7, 10 and 1 are in close proximity to IMD observations for longitude. In case of latitude, the treatments 9, 8, 13, 2, 1, 23, 5 and 20 are similar to treatment 0. After overlooking the inconsistent treatments in all the variables, the treatments 1 (2.25 hPa) and 5 (2.91 hPa) for CP and treatments 1 (2.05 ms^−1^) and 4 (2.04 ms^−1^) for MSW, result in lower values of RMSE respectively (Fig. 1). The time error at landfall is accurately forecasted by treatments 1 and 2 for cyclone Nargis (Fig. 2). Overall, the treatment 1 is consistent in the case of Titli, Fani and Nargis.

**Table 10:** One-way ANOVA for Nargis Longitude.

	Sum of squares	df	Mean square	F	Sig
Between groups	312.626	30	10.421	0.917	0.595
Within groups	5282.540	465	11.360		
Total	5595.167	495

**Table 11:** One-way ANOVA for Nargis latitude.

	Sum of squares	df	Mean square	F	Sig
Between groups	146.103	30	4.870	1.658	0.017
Within groups	1365.529	465	2.937		
Total	1511.632	495

**Table 12:** One-way ANOVA for Nargis CP.

	Sum of squares	df	Mean square	F	Sig
Between groups	21,310.887	30	710.363	2.272	0.000
Within groups	145,366.020	465	312.615		
Total	166,676.907	495

**Table 13:** One-way ANOVA for Nargis MSW.

	Sum of squares	df	Mean square	F	Sig
Between groups	11,155.243	30	371.841	4.899	0.000
Within groups	35,293.356	465	75.900		
Total	46,448.599	495			

## Discussion and conclusion

One way ANOVA has been conducted on the values obtained after the model simulation with thirty combinations of physics schemes (treatments 1–30) along with the IMD observations (treatment 0) for intensity and track variables. The results obtained from the one-way ANOVA shows that the average effect of treatments for all the variables (intensity and track) are significantly different between themselves. The observed significance level is less than α = 0.10 in the case of intensity and track variables for cyclone Titli, which suggests that average effect of all treatments can be differentiated within the considered CI. On the contrary, it is noticeable for cyclone Fani, average effect of all the treatments differs significantly for longitude, CP and MSW. The treatments for latitude variable are similar to one another in the 90% CI. The *p* values for CP and MSW of cyclone Nargis are less than α = 0.10; which implies that all the treatments differ significantly within themselves as well as with the observations. One way analysis of variance (ANOVA) rejects the null hypothesis for latitude whereas it accepts the same for longitude. This signifies that the treatment means are significantly different among themselves for latitude and do not vary in case of longitude. The basic purpose of the study is to identify which treatment combination is close to IMD observations. Therefore, Tukey’s test is used for post-hoc analysis.

Significant difference in mean effect of the treatments concludes that their simulated population means were equal at 90% confidence interval, and it was difficult to distinguish them from one another. Tukey’s post-hoc test is applied individually on the same variables for average effect of all treatments. This post-hoc test uses the “Honest Significant Difference”, a number that represents the distance between groups, to compare every mean with every other treatment mean. Less the difference, the more similar the treatments are to one another. In case of cyclone Titli, Tukey’s test analysis for CP exhibits least differences for treatment 9 and 6 with treatment 0, followed by treatment 12, 16, 13, 14, 20, 5, 4 and 1. Whereas, the treatments 10, 29, 28, 24, 22, 27, 1, 19, 2, 18, 4 and 3 are found to be substantially close to IMD observations for MSW (Table 14b). In case of latitude, the post-hoc analysis reveals that treatment 1, 4, 2, 3, 16 and 23; while for longitude the treatments 1, 12, 28, 14 and 3 are significantly close to treatment 0 respectively (Table 15b). The treatments 9, 6, 12, 14, 16, 13, 20, 5, 13, 10, 29, 28, 24, 22, 27, 19, 18, 28, 16, and 23 are excluded in optimization. These cluster of treatments display considerable difference as a result of Tukey’s test yet, due do the inconsistency in the occurrences all the variables they are eliminated. Out of the consistent cluster of treatments, the treatments 1 and 3 for CP and treatment 1 for MSW show least average RMSE values. The time error at landfall by treatments 1 and 4 are notably close to treatment 0. It can be noticed that treatment 1 consistently exhibits significantly close values to IMD, hence the combination of YSU PBL, Kain-Fritsch CC and Ferrier MP Scheme can be asserted as the optimal combination for cyclone Titli.

**Table 14:** Tukey post-hoc analysis for CP and MSW for cyclone (a) Fani (b) Titli and (c) Nargis.

(a) Fani				(b) Titli				(c) Nargis			
CP	Treatment	MSW	Treatment	CP	Treatment	MSW	Treatment	CP	Treatment	MSW	Treatment
969.572	0	42.206	10	988.478	1	23.128	10	978.375	0	29.406	1
970.281	20	42.400	19	989.006	4	23.291	0	981.203	18	29.637	19
970.425	18	42.725	3	989.006	5	23.422	29	982.206	2	30.906	17
971.369	19	42.956	17	990.650	20	23.461	28	983.189	3	31.5	4
971.394	1	43.825	4	991.300	13	23.628	24	983.725	19	31.694	5
971.402	5	44.594	2	991.572	16	23.656	22	983.796	4	31.987	3
972.097	17	44.675	1	992.722	0	23.872	27	984.350	5	32.131	18
972.631	2	45.010	0	992.806	9	30.922	1	984.409	17	34.006	2
				992.831	6	31.706	19	986.803	1	34.786	0
				992.967	12	32.989	2				
				993.056	14	33.239	18				
						34.100	4				
						34.339	3

**Table 15:** Tukey post-hoc analysis for Longitude and Latitude for cyclone (a) Fani (b) Titli and (c) Nargis.

(a) Fani				(b) Titli				(c) Nargis			
Long	Treatment	Lat	Treatment	Long	Treatment	Lat	Treatment	Long	Treatment	Lat	Treatment
85.285	7	17.677	1	84.752	3	16.467	23	90.331	0	15.500	0
85.326	2	17.728	2	84.809	14	16.636	16	90.734	20	15.781	9
85.351	8	17.750	18	84.995	28	16.955	0	90.770	5	15.840	8
85.535	1	17.806	17	85.036	0	16.955	1	90.994	18	15.854	13
85.560	21	17.872	20	85.036	1	16.986	4	91.066	2	15.869	2
85.568	13	18.098	5	85.045	12	17.127	2	91.193	3	16.334	1
85.735	0	18.129	0			17.154	3	91.197	19	16.336	23
								91.227	4	16.491	5
								91.299	16	16.676	20
								91.301	25		
								91.328	6		
								91.328	7		
								91.336	17		
								91.387	10		
								91.427	1		

The treatments 20 and 18 show close relation with treatment 0 for CP, whereas treatment 1 and 2 are significantly close to treatment 0 for MSW in post-hoc analysis for cyclone Fani. The cluster of treatments 19, 1, 5, 17 and 2 for CP and treatments 4, 17, 3, 19 and 10 for MSW also display significantly closer values to that of IMD observations. The treatments 13, 21, 1, 8, 2 and 7 are considerably close to treatment 0 for latitude whereas, the group of treatments 5, 20, 17, 18, 2 and 1 are substantively similar to treatment 0 for longitude. The treatments other than these four clusters of treatments are excluded at this stage. After the elimination, the treatments which result in significantly lower values of RMSE (CP and MSW) and accuracy in time of landfall are noted. The treatments 1 and 2 display lesser values for RMSE of both CP and MSW. The time of landfall is also precisely captured by treatments 1 and 2 for cyclone Fani.

The analysis of CP for cyclone Nargis displays treatment 18 to be closest to treatment 0, followed by treatment 2, 3, 19, 4, 5 17, and 1 (Table 14c). But it does not imply that 5, 17 and 1 are less significant. For wind intensity, it may be observed that treatment 2 and 18 are closest to observations. Yet, treatments 3, 4, 9, 1 and 17 are considerably comparable to treatment 0. The treatments 20 and 5 for longitude and, treatments 9 and 8 for latitude have minimal difference with treatment 0. The group of treatments 18, 2, 3, 19, 4, 16, 25, 6, 17, 7, 10 and 1 are also in close proximity to IMD observations for longitude. Whereas, for latitude, the treatments 13, 2, 1, 23, 5 and 20 are similar to treatment 0. At this stage, the treatments which are inconsistent in all the variables are eliminated. Out of the remaining treatments, the treatments 1 and 5 for CP and treatments 1 and 4 for MSW are having average RMSE significantly close to IMD observations respectively. Accuracy in time of landfall is another vital factor to be considered while optimization. The treatments 1 and 2 have provided accuracy in the time error at landfall for cyclone Nargis. Yet, treatment 2 is eliminated as it does not comply with the RMSE in comparison to treatment 1. This makes treatment 1 the optimal scheme combination for cyclone Nargis. The present results strongly corroborate with the earlier studies on tropical cyclone simulations using the combination YSU PBL, KF CC and Ferrier MP^9,10,22^. Overall, based on the elimination process treatment 1 (YSU–KF–Ferrier) exhibits significantly close and persistence with the IMD observations.

## Methods

The process of scrutinization of different combination of three physics schemes of WRF model is discussed in this section. The scheme combinations are accessed with respect to the intensity variables (CP; MSW) and track (longitude, latitude) individually on three cyclonic cases over the Bay of Bengal. Here, the tropical cyclone parameters (CP, MSW, longitude, latitude) represent the factors, and the scheme combination represent the treatments. The intensity variables are considered as they play an important role in determining the strength and stage of tropical cyclone whereas the position (longitude, latitude) is required to accurately depict the movement of cyclone. These four factors help the policy makers to predict the area of landfall, and the damage as a result of this catastrophe.

The 0th treatment is the IMD observation which is followed by the model results obtained from 30 combinations (treatments 1–30), which accounts to 31 treatments. The optimization algorithm to obtain the optimal physics scheme combination employed in the study are described as follows.

1. Firstly, one-way analysis of variance (ANOVA) is applied on 31 treatment combinations. Then significant difference has been obtained among these treatments for each variable (CP, MSW, longitude and latitude) corresponding to each cyclone; and it is found that there is a significant difference among the 31 treatment combinations.
2. Since there was a significant difference among the 31 different treatments so, pair of treatments which are significantly different are found out through Tukey’s test. This test has been applied individually on four variables of three cyclones respectively.
3. The treatments (treatments 1–30) which were consistently having no significant difference with IMD observations (treatment 0) were then tested for RMSE (CP and MSW). Average RMSE was computed over the whole TC period and the group of treatments which have significantly minimum values are found. Further, landfall time was also tested, and the treatments have been selected which were nearer to IMD observations.
4. Consistence occurrence of the treatment in all the above steps is treated as the optimal treatment.

One-way ANOVA is applied to compare two treatments, as it is necessary to identify whether the thirty-one treatments differ from each other with respect to the individual variables (CP, MSW, Longitude and Latitude). The statistical null hypothesis *H*_0_ and alternative hypothesis *H*_1_ are described as follows:

*H*_*0*_ The average effect all the 31 treatments are similar to one another.

*H*_*1*_ The average effect all the 31 treatments are significantly different.

To test the null hypothesis, One-way Analysis of Variance (ANOVA) is applied for all the factors and on all three tropical cyclone cases (Nargis, Titli and Fani). ANOVA uses F-test for statistical significance, which allows for comparison of multiple means at once. Thus, the error is computed for the whole set of comparisons rather than individual. If any of the treatment means is significantly different from the overall mean, then the null hypothesis is rejected. The hypothesis is checked at 90% confidence interval (CI). The *p* value is then compared to the critical value *p*_α_ at degrees of freedom *(k, N − k)*, where *α* is the significance level i.e., 0.10; *k* is the number of treatments, and *N* is the total sample size. If the *p* value is less than *p*_α_ *(k, N − k)*, then the null hypothesis is rejected.

If the null hypothesis is rejected then ANOVA identifies whether there are differences among the levels of individual variables viz. differences among the treatments for individual variables, but it will not specify which differences are significant. With the purpose to identify the difference between the treatments, Tukey’s Post-Hoc test is applied to test the following hypothesis.

*H*_*0*_ There is no significant difference between the average effect of any two treatments.

*H*_*1*_ There is a significant difference between the average effect of any two treatments.

Tukey’s test runs pairwise comparisons among each of the groups, and uses a conservative error estimate to find the groups which are statistically different from one another. The output of the Tukey’s test provides a table where treatments are arranged in order of error, from where one can identify the treatment closest to 0th viz. IMD observations. This post-hoc analysis is conducted respectively for all the four variables corresponding to the three cyclone cases.

From the results obtained from the Tukey’s test, a cluster of different treatments close to observations are obtained for individual cyclonic cases with their respective variables. Treatments that are identified close to observed values need to be checked for optimality. Since, inaccuracy in forecasting of landfall time error and its intensity could result in devastation of life and infrastructure over a region thus, the RMSE computed for the CP, MSW, and the time of landfall are looked into for respective treatments. The treatment which is consistently close to IMD observations in the cyclones is suggested as the optimum treatment through elimination process for the simulation of tropical cyclones over the Bay of Bengal (“Supplementary information”).

### Statistical analysis

Statistical analyses are conducted using SPSS, version 22.0 (SPSS, Inc., Chicago, IL, United States). One way ANOVA is used to check the significance of observations obtained from model simulations with various combination of physics schemes hereafter termed as treatments. Results from ANOVA are reported with 90% confidence intervals and are deemed significant at *p* < 0.10. Tukey’s post-hoc test is further used to test the significant difference between any two treatment means.

## Supplementary Information

Supplementary Information.
