# Supplementary material for: Optimization of physical schemes in WRF model on cyclone simulations over Bay of Bengal using one-way ANOVA and Tukey’s test
Source: Sci Rep. 2021 Dec 24;11:24412. doi: 10.1038/s41598-021-02723-z (PMC8709857; doi:10.1038/s41598-021-02723-z)
Supplement: Supplementary file 1 — Supplementary Information. [file 41598_2021_2723_MOESM1_ESM.docx]

**Supplementary Material**

| **Table 16: Tukey post-hoc analysis for CP and MSW for cyclone a) Fani b) Titli and c) Nargis** | | | | | | | | | | | | | |
| --- | --- | --- | --- | --- | --- | --- | --- | --- | --- | --- | --- | --- | --- |
| 1. Fani | | | | 1. Titli | | | | | 1. Nargis | | | | |
| CP | treatment | MSW | treatment |  | CP | treatment | MSW | treatment |  | CP | treatment | MSW | treatment |
| 969.5719 | 0 | 35.0062 | 15 |  | 981.8861 | 3 |  | 30 |  | 978.375 | 0 |  | 28 |
| 970.2813 | 20 | 35.1 | 30 |  | 981.9333 | 19 |  | 15 |  | 981.2031 | 18 |  | 27 |
| 970.425 | 18 | 35.9125 | 21 |  | 983.0889 | 18 |  | 26 |  | 982.2063 | 2 |  | 29 |
| 971.3688 | 19 | 36.9563 | 26 |  | 983.1222 | 2 |  | 21 |  | 983.1894 | 3 |  | 12 |
| 971.3938 | 1 | 38.1062 | 23 |  | 983.7283 | 17 |  | 25 |  | 983.725 | 19 |  | 26 |
| 971.4025 | 5 | 38.1187 | 8 |  | 988.4778 | 1 | 23.1278 | 10 |  | 983.7956 | 4 |  | 30 |
| 972.0969 | 17 | 38.2937 | 11 |  | 989.0056 | 4 | 23.2909 | 0 |  | 984.35 | 5 |  | 14 |
| 972.6313 | 2 | 38.3125 | 28 |  | 989.0056 | 5 | 23.4222 | 29 |  | 984.4094 | 17 |  | 15 |
| 973.0938 | 3 | 38.525 | 27 |  | 990.65 | 20 | 23.4611 | 28 |  | 986.8031 | 1 |  | 11 |
| 973.7063 | 4 | 38.5375 | 13 |  | 991.3 | 13 | 23.6278 | 24 |  | 988.1625 | 20 |  | 13 |
| 973.9469 | 29 | 38.7063 | 12 |  | 991.5722 | 16 | 23.6556 | 22 |  | 989.2563 | 16 |  | 24 |
| 974.88 | 10 | 39.2438 | 22 |  | 992.7222 | 0 | 23.8722 | 27 |  | 994.6062 | 6 |  | 23 |
| 975.1938 | 16 | 39.4438 | 29 |  | 992.8056 | 9 | 24.0667 | 23 |  | 994.6062 | 7 |  | 25 |
| 975.725 | 30 | 39.5219 | 25 |  | 992.8306 | 6 | 24.3667 | 11 |  | 995.3625 | 11 |  | 22 |
| 975.8187 | 12 | 39.5687 | 7 |  | 992.9667 | 12 | 24.3672 | 13 |  | 995.65 | 15 |  | 21 |
| 976.2656 | 25 | 39.8813 | 14 |  | 993.0556 | 14 | 25.0056 | 12 |  | 996.3812 | 12 |  | 8 |
| 976.5375 | 27 | 40.1062 | 16 |  | 993.2972 | 7 | 25.4111 | 14 |  | 996.4625 | 9 |  | 10 |
| 976.5938 | 28 | 40.15 | 24 |  | 993.3167 | 11 | 25.6278 | 6 |  | 996.55 | 14 | 23.9812 | 7 |
| 976.6375 | 15 | 40.42 | 6 |  | 994.1722 | 8 | 25.9111 | 20 |  | 996.725 | 10 | 23.9875 | 9 |
| 976.7312 | 26 | 41.2188 | 9 |  | 995.05 | 27 | 26.0111 | 8 |  | 996.8125 | 13 | 25.3938 | 6 |
| 977.1313 | 24 | 41.8062 | 20 |  | 995.1194 | 10 | 26.2111 | 7 |  | 996.825 | 8 | 26.2062 | 16 |
| 977.2062 | 11 | 41.9563 | 5 |  | 995.2139 | 23 | 26.7167 | 9 |  | 997.025 | 30 | 27.8437 | 20 |
| 979.2312 | 14 | 42.1563 | 18 |  | 995.2222 | 28 | 27.6167 | 16 |  | 997.35 | 21 | 29.4063 | 1 |
| 980.6406 | 13 | 42.2063 | 10 |  | 995.3167 | 29 | 27.9944 | 5 |  | 997.35 | 22 | 29.6375 | 19 |
| 981.1875 | 9 | 42.4 | 19 |  | 996.55 | 25 | 30.4222 | 17 |  | 997.5438 | 25 | 30.9062 | 17 |
| 981.3125 | 6 | 42.725 | 3 |  | 996.5861 | 22 | 30.9222 | 1 |  | 998.0875 | 26 | 31.5 | 4 |
| 982.225 | 22 | 42.9563 | 17 |  | 996.6828 | 24 | 31.7056 | 19 |  | 998.2125 | 24 | 31.6937 | 5 |
| 982.975 | 23 | 43.825 | 4 |  | 997.0417 | 26 | 32.9889 | 2 |  | 998.4219 | 23 | 31.9875 | 3 |
| 983.4656 | 7 | 44.5938 | 2 |  | 997.6861 | 21 | 33.2389 | 18 |  | 998.6438 | 27 | 32.1312 | 18 |
| 984.2 | 21 | 44.675 | 1 |  | 997.8667 | 15 | 34.1 | 4 |  | 999.7375 | 28 | 34.0063 | 2 |
| 0.854 | 8 | 45.01 | 0 |  | 999.6639 | 30 | 34.3389 | 3 |  | 999.875 | 29 | 34.7863 | 0 |

| **Table 17: Tukey post-hoc analysis for Longitude and Latitude for cyclone a) Fani b) Titli and c) Nargis** | | | | | | | | | | | | | |
| --- | --- | --- | --- | --- | --- | --- | --- | --- | --- | --- | --- | --- | --- |
| 1. Fani | | | | 1. Titli | | | | | 1. Nargis | | | | |
| Longitude | treatment | Latitude | treatment |  | Longitude | treatment | Latitude | treatment |  | Longitude | treatment | Latitude | treatment |
|  | 20 | 15.7085 | 14 |  | 82.7111 | 20 |  | 26 |  | 89.2031 | 30 | 14.7969 | 12 |
|  | 5 | 15.8432 | 12 |  | 82.8061 | 5 |  | 5 |  | 89.2913 | 11 | 14.8550 | 14 |
| 84.5323 | 19 | 15.9915 | 11 |  | 83.0183 | 9 |  | 9 |  | 89.3600 | 15 | 14.8713 | 27 |
| 84.6006 | 25 | 16.0097 | 13 |  | 83.3744 | 7 |  | 20 |  | 89.7175 | 27 | 14.9469 | 11 |
| 84.6712 | 28 | 16.1071 | 26 |  | 83.3939 | 30 | 15.6494 | 7 |  | 89.8756 | 14 | 14.9656 | 29 |
| 84.6876 | 24 | 16.1544 | 9 |  | 83.4529 | 24 | 15.7883 | 30 |  | 89.8781 | 28 | 15.0325 | 28 |
| 84.7215 | 18 | 16.2165 | 8 |  | 83.5850 | 10 | 15.8578 | 10 |  | 89.8856 | 12 | 15.1544 | 26 |
| 84.7359 | 29 | 16.3012 | 7 |  | 83.6717 | 25 | 15.8894 | 14 |  | 89.9444 | 29 | 15.2019 | 15 |
| 84.7929 | 30 | 16.3094 | 27 |  | 83.7667 | 21 | 15.9311 | 21 |  | 90.0331 | 26 | 15.3106 | 30 |
| 84.8000 | 17 | 16.3382 | 29 |  | 83.7722 | 22 | 15.9722 | 15 |  | 90.3313 | 0 | 15.5000 | 0 |
| 84.8632 | 9 | 16.3414 | 6 |  | 83.8039 | 15 | 16.0375 | 22 |  | 90.7344 | 20 | 15.7813 | 9 |
| 84.8644 | 27 | 16.3864 | 15 |  | 84.1517 | 16 | 16.0388 | 24 |  | 90.7700 | 5 | 15.8400 | 8 |
| 84.8818 | 14 | 16.5282 | 28 |  | 84.1989 | 6 | 16.0844 | 8 |  | 90.9938 | 18 | 15.8544 | 13 |
| 84.9203 | 15 | 16.6006 | 10 |  | 84.2022 | 8 | 16.0844 | 25 |  | 91.0663 | 2 | 15.8694 | 2 |
| 84.9426 | 12 | 16.6135 | 30 |  | 84.2244 | 19 | 16.0911 | 29 |  | 91.1931 | 3 | 15.8963 | 6 |
| 84.9900 | 26 | 16.7056 | 21 |  | 84.3350 | 4 | 16.1506 | 12 |  | 91.1969 | 19 | 15.8963 | 7 |
| 85.0385 | 10 | 16.7641 | 23 |  | 84.3383 | 23 | 16.2272 | 27 |  | 91.2269 | 4 | 15.9181 | 4 |
| 85.0724 | 11 | 16.7982 | 25 |  | 84.3892 | 17 | 16.2783 | 6 |  | 91.2994 | 16 | 16.0138 | 22 |
| 85.1000 | 3 | 16.9547 | 24 |  | 84.4411 | 26 | 16.3122 | 11 |  | 91.3006 | 25 | 16.0241 | 3 |
| 85.1000 | 4 | 16.9800 | 22 |  | 84.5900 | 2 | 16.4094 | 28 |  | 91.3281 | 6 | 16.0319 | 21 |
| 85.1129 | 22 | 17.6312 | 3 |  | 84.6400 | 27 | 16.4378 | 13 |  | 91.3281 | 7 | 16.1256 | 19 |
| 85.1694 | 23 | 17.6312 | 4 |  | 84.6806 | 29 | 16.4672 | 23 |  | 91.3356 | 17 | 16.1669 | 17 |
| 85.1965 | 16 | 17.6554 | 19 |  | 84.6828 | 18 | 16.6361 | 16 |  | 91.3875 | 10 | 16.1763 | 24 |
| 85.2488 | 6 | 17.6729 | 16 |  | 84.7522 | 3 | 16.9553 | 0 |  | 91.4275 | 1 | 16.1844 | 18 |
| 85.2853 | 7 | 17.6771 | 1 |  | 84.8094 | 14 | 16.9553 | 1 |  | 91.5106 | 22 | 16.2588 | 16 |
| 85.3256 | 2 | 17.7282 | 2 |  | 84.9950 | 28 | 16.9861 | 4 |  | 91.5787 | 13 | 16.3006 | 25 |
| 85.3506 | 8 | 17.7503 | 18 |  | 85.0358 | 0 | 17.1267 | 2 |  | 91.6050 | 8 | 16.3163 | 10 |
| 85.5347 | 1 | 17.8059 | 17 |  | 85.0358 | 1 | 17.1544 | 3 |  | 91.6294 | 23 | 16.3344 | 1 |
| 85.5600 | 21 | 17.8724 | 20 |  | 85.0450 | 12 | 17.1947 | 19 |  | 91.6300 | 21 | 16.3363 | 23 |
| 85.5685 | 13 | 18.0982 | 5 |  | 85.0711 | 13 | 17.2247 | 17 |  | 91.6738 | 9 | 16.4913 | 5 |
| 85.7353 | 0 | 18.1294 | 0 |  | 85.1456 | 11 | 17.3883 | 18 |  | 91.6994 | 24 | 16.6756 | 20 |
